# The Time of Maximum Post-Ischemic Hyperperfusion Indicates Infarct Growth Following Transient Experimental Ischemia

**DOI:** 10.1371/journal.pone.0065322

**Published:** 2013-05-31

**Authors:** Susanne Wegener, Judith Artmann, Andreas R. Luft, Richard B. Buxton, Michael Weller, Eric C. Wong

**Affiliations:** 1 Department of Neurology, University Hospital Zurich, Zurich, Switzerland; 2 Department of Radiology, University of California San Diego, San Diego, California, United States of America; 3 Department of Psychiatry, University of California San Diego, San Diego, California, United States of America; University of Queensland, Australia

## Abstract

After recanalization, cerebral blood flow (CBF) can increase above baseline in cerebral ischemia. However, the significance of post-ischemic hyperperfusion for tissue recovery remains unclear. To analyze the course of post-ischemic hyperperfusion and its impact on vascular function, we used magnetic resonance imaging (MRI) with pulsed arterial spin labeling (pASL) and measured CBF quantitatively during and after a 60 minute transient middle cerebral artery occlusion (MCAO) in adult rats. We added a 5% CO_2_ - challenge to analyze vasoreactivity in the same animals. Results from MRI were compared to histological correlates of angiogenesis. We found that CBF in the ischemic area recovered within one day and reached values significantly above contralateral thereafter. The extent of hyperperfusion changed over time, which was related to final infarct size: early (day 1) maximal hyperperfusion was associated with smaller lesions, whereas a later (day 4) maximum indicated large lesions. Furthermore, after initial vasoparalysis within the ischemic area, vasoreactivity on day 14 was above baseline in a fraction of animals, along with a higher density of blood vessels in the ischemic border zone. These data provide further evidence that late post-ischemic hyperperfusion is a sequel of ischemic damage in regions that are likely to undergo infarction. However, it is transient and its resolution coincides with re-gaining of vascular structure and function.

## Introduction

“Luxury perfusion syndrome” is a term coined by Niels Lassen in 1966. It refers to a state of overabundant cerebral blood flow (CBF) relative to the metabolic needs of the brain tissue, as is observed within seconds after reopening of a formerly occluded artery in experimental stroke models [1]. Early post-ischemic hyperperfusion is usually abrupt, lasts only for minutes to a few hours and is closely related to severity and length of prior ischemia [2], [3]. High CBF in this early stage after ischemia has been correlated to more severe neuronal damage and worse outcome, mediated, in part, by the overproduction and release of toxic free radicals [4], [5].

Evidence for a similar phenomenon in humans comes from clinical studies, where CBF was increased above normal in some patients after vessel recanalization [6], [7], [8], [9]. Although there is consensus that at least partial recanalization is a prerequisite for hyperperfusion after stroke, the incidence as well the meaning of hyperperfusion for patient recovery have remained controversial [8], [10]. It may be assumed, however, that the mechanisms of early post-ischemic hyperperfusion in animals are distinct from late hyperperfusion observed in patients up to weeks after stroke [11]. Animal stroke models allow for the observation of blood flow over time following a controlled stroke and reperfusion paradigm. Perfusion-weighted MRI has been frequently applied as a non-invasive method to measure CBF [12], [13]. However, only very few data exist about CBF at more chronic time points after experimental ischemia, which would better resemble the clinical observation times of hyperperfusion in stroke patients [14], [15], [16]. We used a rat stroke model and quantified CBF with pulsed arterial spin labeling (pASL) MRI. Our goal was to find out how CBF is maintained at different time intervals after reperfusion and how the capacity for vasodilation recovers in the ischemic area.

## Materials and Methods

### Animals and experimental protocol

All experiments were performed in accordance with the National Institutes of Health animal protection guidelines and approved by the Institutional Animal Care and Use Committee, University of California, San Diego (Protocol number S04192).

In this study, 14 adult male Wistar rats (280–320 g body weight) were used. For MRI and surgery, animals were anesthetized using facemask inhalation of 1.5–2.5% isoflurane in a 2∶1 N_2_O∶O_2_ atmosphere. Temperature was maintained at 37°C by a feedback-controlled heating pad.

Six animals were subjected to a single MRI session for CBF quantification under the isoflurane anesthesia described above (referred to as “air”) and with an additional 5% CO_2_ challenge (referred to as 5% CO_2_). Using arterial blood gas analyses we found in a separate set of animals (using the identical set-up and performed within 6 months of the experiments presented here) that these anesthesia conditions resulted in normoxia (“air”) and hypercapnia (5% CO_2_) followed by a robust increase in CBF during hypercapnia [17]. In 8 rats, transient ischemia was induced for 60 minutes by MCAO using the intraluminal thread model [18]. Briefly, a 4-0 silicone-coated filament was introduced into the common carotid artery and advanced approximately 16 to 20 mm from the carotid bifurcation until a slight resistance was felt. The thread was left in place for 60 min. During that time and without discontinuation of anesthesia, the animal was placed in the custom-built cradle for the first (day0) MRI examination. After 60 minutes, the thread was withdrawn and the animal allowed to recover. Animals were re-anesthetized for repetitive MRI after MCAO: MRI assessment was performed at the following time points: day 0 (d0), d1, d4, and d14 after MCAO. For induction of hypercapnia, the isoflurane concentration was kept constant while the inhaled gas composition was changed to 5% CO_2_ in medical air. A 2 minute adjustment was allowed between switching the gas and initiating data acquisition. The overall duration of isoflurane anesthesia for the MRI scans on d1 to d14 was 45 minutes. Before MRI, stroke-induced functional deficits were assessed using an 18-point composite neurological score [19]. The test incorporated the observation of (1) spontaneous activity, (2) symmetry in limb movement, (3) forepaw outstretching, (4) climbing, (5) body proprioception, and (6) response to vibrissae touch, each scored with a maximum of three points, so that 18 points indicated no neurological deficit. After the last MRI, animals were killed by an overdose of pentobarbital and subjected to transcardial perfusion fixation with 0.1 mol/L phosphate-buffered saline (PBS), followed by 4% formalin in phosphate buffer. Brains were extracted for histological analysis.

### MRI experiments

MRI experiments were carried out on a 3 T GE Signa Excite whole-body system with a body transmit coil and a custom-built passively decoupled single-loop receive-only head coil of 3 cm diameter [20]. For perfusion imaging, we used multislice flow-sensitive alternating inversion recovery (FAIR) pulsed arterial spin labeling (pASL) with the QUIPSS II modification to minimize the effects of transit delays [21]. Imaging parameters were as follows: multi-shot spiral; slice thickness: 2 mm (three slices); gap: 1 mm; field of view: 4×4 cm; matrix: 64×64; flip angle: 90°, number of interleaves: 8, number of repetitions: 12; TE: 4.3 ms; TI_1_/TI_2_: 700/1250 ms gap between tagging and imaging region: 5 mm. In-plane saturation was applied immediately after the preparatory inversion pulse for background suppression. Artefacts from pulsations of the carotid arteries below the brain were suppressed with a spatial saturation pulse. The image acquisition was immediately followed by a global saturation (PostSat) to reset the blood signal. For details of equilibrium magnetization reference scan and coil sensitivity profile estimation see [20].

CBF was calculated from the signal difference ΔS between tag and control ASL images according to:
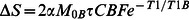
(1)where α is the tagging efficiency, *M*_0*B*_ is the MRI signal from a voxel full of arterial blood, τ is the temporal width of the blood bolus that reaches the region of interest, and *T*1*B* is the T1 of blood. 2α*M*_0*B*_ is the initial magnetization difference between tagged and control blood, the product τ*CBF* is the amount of tagged blood that flows into the region of interest (ROI), and the exponential factor reflects T1relaxation of the tag.

In addition, anatomical images (T2-weighted, 256×256), as well as T1 and T2 maps were acquired with the same slice prescription as the ASL experiment.

### MRI data processing and statistical analyses

For MRI data processing, images from all animals per group were first co-registered and then averaged using codes written in Matlab (Mathworks, Natick, MA, USA) software. Group values given in this study were obtained by ROI analysis on the co-registered and averaged MR images. Vasoreactivity (VR) maps were generated by subtracting the CBF map acquired in air from the one acquired in 5% CO_2_. To define voxels with hyperperfusion, the mean signal and standard deviation of the contralateral hemisphere (excluding the ventricles) were obtained on CBF maps. Voxels with CBF values above this mean plus two standard deviations were considered as “hyperperfused”. Similarly, voxels with T2 values above the mean and two standard deviations of the contralateral hemisphere were assigned to the final infarct. Statistical analyses were conducted using SPSS v12.0 for Windows. All values were reported as mean ± standard deviation. The area of RECA-positive signal within the ROIs was compared by independent *t*-test. CBF values between ischemic and contralateral voxels were compared with a paired *t*-test. The Pearson correlation coefficient was used for correlation analyses between MRI parameters. A Repeated Measures General Linear Model (GLM) Analysis was conducted for the comparison of ipsi-and contralateral CBF values. *P* values below 0.05 were considered significant.

### Histology

After perfusion fixation, brains were removed, immersed in ice-cold 4% formalin in phosphate buffer overnight and transferred to 30% sucrose solution for at least 3 days. Coronal 40-µm-thick sections were cut on a freezing microtome (Leica, Nussloch, Germany). Premounted sections were stained with hematoxylin eosin (HE). Immunofluorescence staining of endothelial cells was performed using mouse anti-rat endothelial cell antigen (RECA-1; 1∶400; AbD Serotec) and fluorescein (FITC) -labeled donkey anti-mouse secondary antibody (1∶250; Dianova).

For analysis of RECA -staining, slices were acquired from a single staining session and photographed under a 20× objective of an epifluorescence microscope, while fluorescence gain parameters were kept constant. FITC - fluorescence signal was quantified using ImageJ software (NIH Bethesda, MD) within a 3×3 mm region of interest (ROI) positioned within the subcortical border zone of the lesion and the homologous contralateral area. The ipsilateral signal density was compared to the contralateral ROI.

## Results

**Figure 1:**
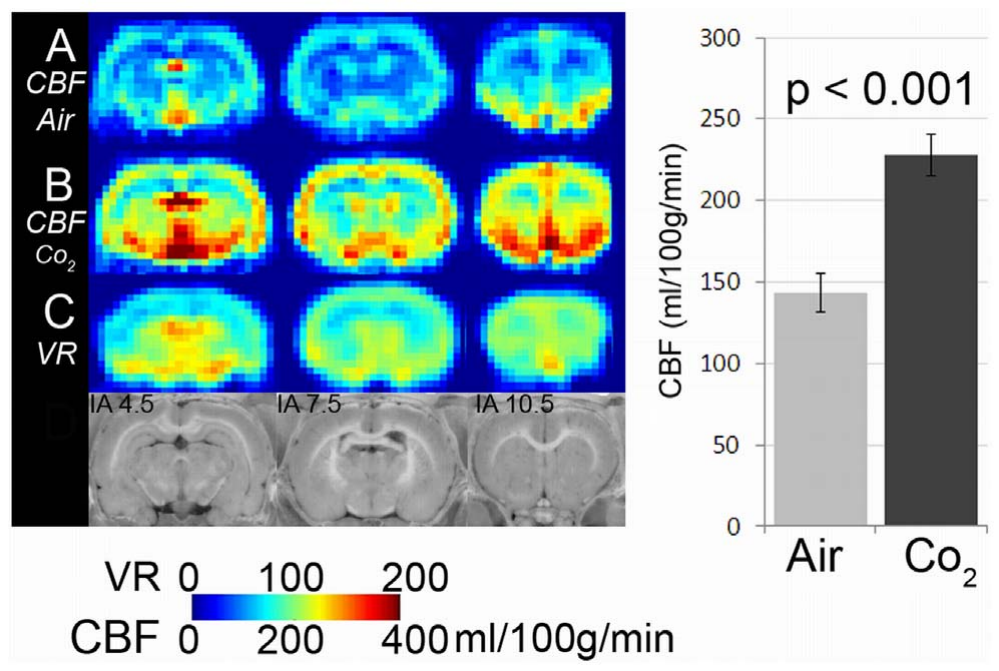
Assessment of vasoreactivity using 5% CO_2_. CBF maps from six healthy animals were averaged. A: averaged CBF map during inhalation anaesthesia with air and B: with 5% CO_2_. C: Vasoreactivity map (VR) obtained by subtracting A from B. D: Anatomical location of brain slices analyzed in MRI with respect to intraaural (IA) line. The graph on the right side displays whole brain CBF values measured with air or CO_2_.

In a first step, CBF was determined in healthy adult Wistar rats (n = 6) under normal (breathing air) and high-flow (breathing 5% CO_2_) conditions. Hypercapnia resulted in a robust increase in CBF throughout the brain, with an average of 58.5% (Figure 1).

**Figure 2:**
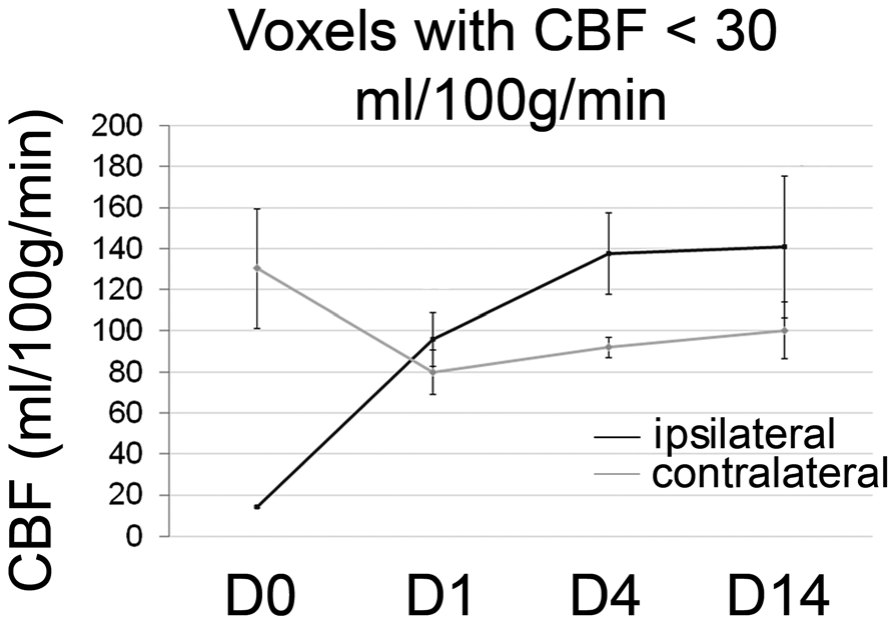
CBF in ischemic voxels over time. Black line: CBF changes within voxels with intra-ischemic CBF of <30 ml/100 g/min between d 0 (D0; during vascular occlusion) up to d14 after MCAO. Grey line: contralateral CBF values from the same animals. Mean ± SD values are shown. The GLM analysis for repeated measures demonstrated a significant effect of the within-subject factor “time” (p = 0.03), but not of the between-subject factor ipsi- or contralateral side (p = 0.7), but for the interception of the two factors (p<0.0001).

**Figure 3:**
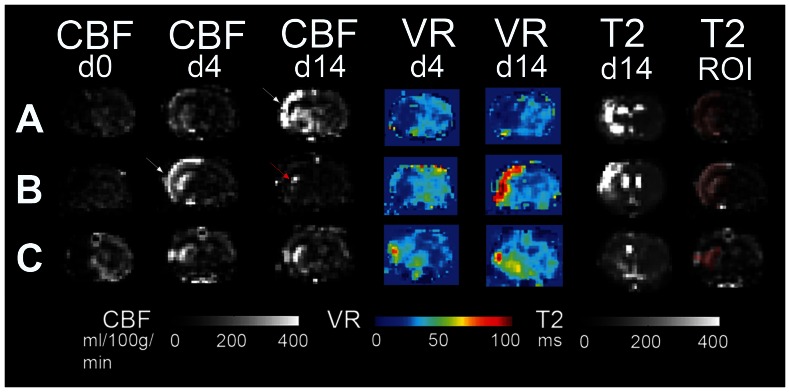
Patterns of hyperperfusion and changes in vasoreactivity. Three representative image series (slice IA 7.5 mm) from animals with A: large cortico-subcortical., B: moderate cortico-subcortical and C: predominantly subcortical infarction. CBF maps on days 0, 4 and 14; along with vasoreactivity (VR) - maps and T2 maps (from d4 and 14/d14) are shown. On the right, the masked infarct area as extracted from D14 T2 maps is overlaid onto the CBF D4 map to show the spatial overlap between hyperperfusion and final infarct. White arrow: points to maximum of hyperperfusion, which occurs at different time points in A) and B). Red arrow in B): when the amount of hyperperfusion has declined, remaining voxels with hyperperfusion are localized more to the infarct border.

**Figure 4:**
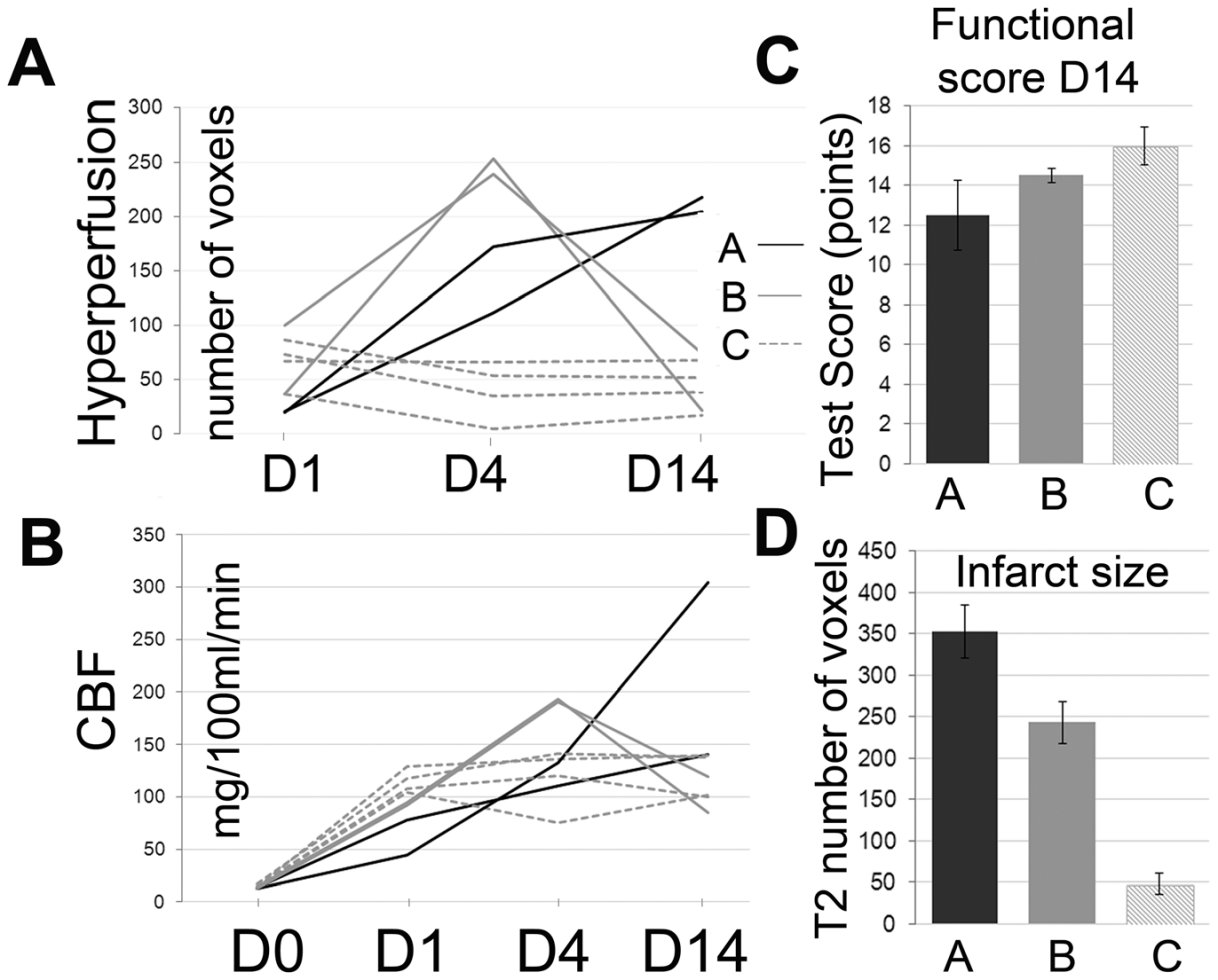
Hyperperfusion and CBF in individual animals. 4A: number of hyperperfused voxels on days 1, 4 and 14 (D1, D4, D14). The three infarct types (A, B, C) show differences in the number of hyperperfused voxels over time. 4B: absolute CBF values (given in mg/100 ml/min) within ischemic voxels (<30 mg/100 ml/min on d0) over time (D0, D1, D4, D14). 4C: Mean functional test scores on D 14 (higher scores indicate less functional impairment) show a gradual increase between infarct types (A, B, C); 4D: Infarct size determined from D14 T2-maps in the three different infarct types.

60 min of MCAO resulted in a profound drop in CBF within the ischemic hemisphere. We grouped voxels displaying a CBF of <30 ml/100 g/min on d0 to follow tissue with severely compromised perfusion over the course of reperfusion and beyond (Figure 2). On day 1 after MCAO, CBF within the d0-defined core voxels had recovered and was on average above contralateral CBF values. Towards d4, CBF rose further above values derived from the contralateral hemisphere. Voxels with an increase in CBF of more than two standard deviations above contralateral were classified as “hyperperfused”. Hyperperfusion evolved in different time courses after MCAO when individual animals were analyzed. Overall, we observed three patterns of hyperperfusion after MCAO (Table 1, Figures 3 and 4A). In large hemispheric cortico-subcortical strokes (type A, n = 2), post-stroke hyperperfusion was first visible at d4 after MCAO, further increasing towards d14. Animals with moderate cortico-subcortical strokes (type B, n = 2) showed a few voxels with hyperperfusion already on d1, with a maximum on d4. Hyperperfusion was clearly remittent towards d14. Predominantly subcortical infarctions (type C, n = 4) already reached the maximum of hyperperfusion on d1, remaining fairly stable or decreasing slightly until d14. To analyze vasodilation as an indicator of vasoreactivity, in addition to standard air conditions, CBF was measured during the application of a 5% CO_2_-challenge on d4 and 14 (Figure 3). In type A strokes, vasoreactivity was depressed on d4 and 14. In type B strokes, vasoreactivity was depressed on d4, but overshooting on d14. Vasoreactivity was either not affected at all or increased in punctate areas within the ischemic lesion on d4, more pronounced on d14, in type C strokes. The final infarct size - determined from T2 maps at day 14 - had a similar distribution as the region of hyperperfusion at its maximum (Figure 3: T2-ROI). The number of hyperperfused voxels on d4 was different between infarct types (Figure 4A) and correlated with final infarct size determined from T2w-MRI on d14 (r = 0.76; p = 0.028); the correlation was even stronger when the number of voxels at the time of maximal hyperperfusion (d1, d4 or d14) were correlated with T2-lesion size (r = 0. 88; p = 0.004). The absolute CBF values within the hyperperfused area could not be used to discriminate infarct type (A, B, C) at any observation time point (Figure 4B). The maximal number of voxels with hyperperfusion correlated with the number of voxels with a severe perfusion restriction (CBF<30 ml/100 g/min) on d0 (r = 0.72; p = 0.04), but not with functional outcome on d14 (r = −0.49). The number of hyperperfused voxels on d14 was a better indicator for functional outcome (r = −0.61), however, this did not reach significance (p = 0.1). In line with this finding, the increment in mean functional test score from type A and B to type C strokes (Figure 4C) was not strong enough to discriminate the three infarct types; whereas T2 lesion size was (Figure 4D).

**Table 1:** Lesion characteristics and functional test scores in individual animals.

	CBF<30 d0	HP d1	HP d4	HP d14	T2 d14	VR+	functional	Stroke
**No1**	187	21	112	218	398	0	10	A
**No2**	119	67	66	68	83	1	14	C
**No3**	55	37	5	17	32	1	18	C
**No4**	120	37	253	22	278	1	14	B
**No5**	150	20	172	204	308	0	15	A
**No6**	145	87	54	52	50	0	16	C
**No7**	185	100	239	74	208	1	15	B
**No8**	92	73	35	39	28	0	14	C

Values from all 8 animals (No1 to No8) are shown. CBF<30 d0: Numbers of voxels with CBF below 30 ml/100 g/min on d0 (considered severely ischemic). HP: hyperperfusion on d 1, 4 and 14. T2 d14: number of voxels of the final infarct which was taken from T2 maps on d 14 as number of voxels with a T2 above the mean and 2 standard deviations of contralateral. VR+ refers to presence of increased vasoreactivity (overshooting CBF response to CO_2_-challenge) on d14. Functional score on d14 is shown (maximum 18 points, minimum 3 points). Stroke was categorized as large cortico-subcortical (A), moderate cortico-subcortical (B) or small, predominantly subcortical (C).

**Figure 5:**
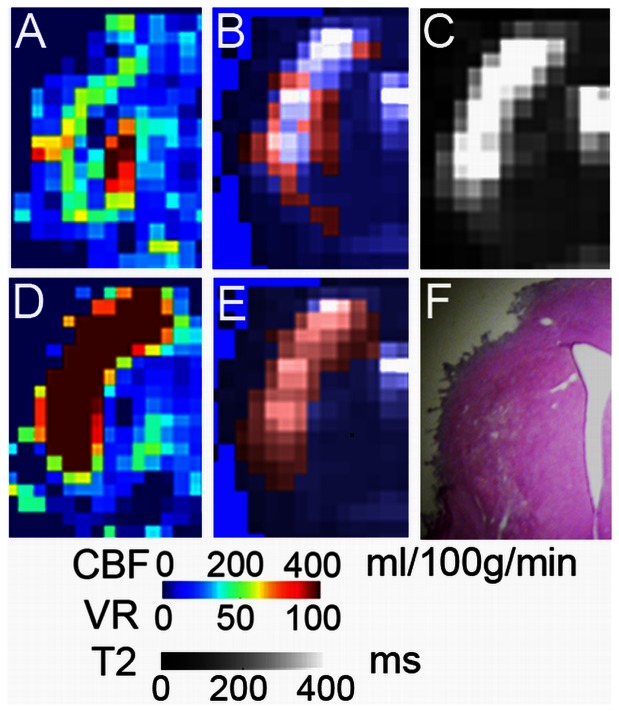
Distribution of voxels with increased d14 vasoreactivity. (A) Infarct region of one animal on d14 - CBF map with hyperperfused voxels (B, shown in red) overlaid onto the d14 - T2 map (B and C). These voxels were localized to the infarct border. (D) Day 14 – vasoreactivity (VR) map. Voxels with increased vasoreactivity are shown in red and overlaid on the T2 map: E. Hematoxylin-eosin stained section of the corresponding region in F.

Voxels in which increased vasoreactivity was observed on d14 mapped to the region of infarct on T2-weighted images (Figure 5). At that time, the remaining voxels with hyperperfusion were localized more around the infarct border zone.

**Figure 6:**
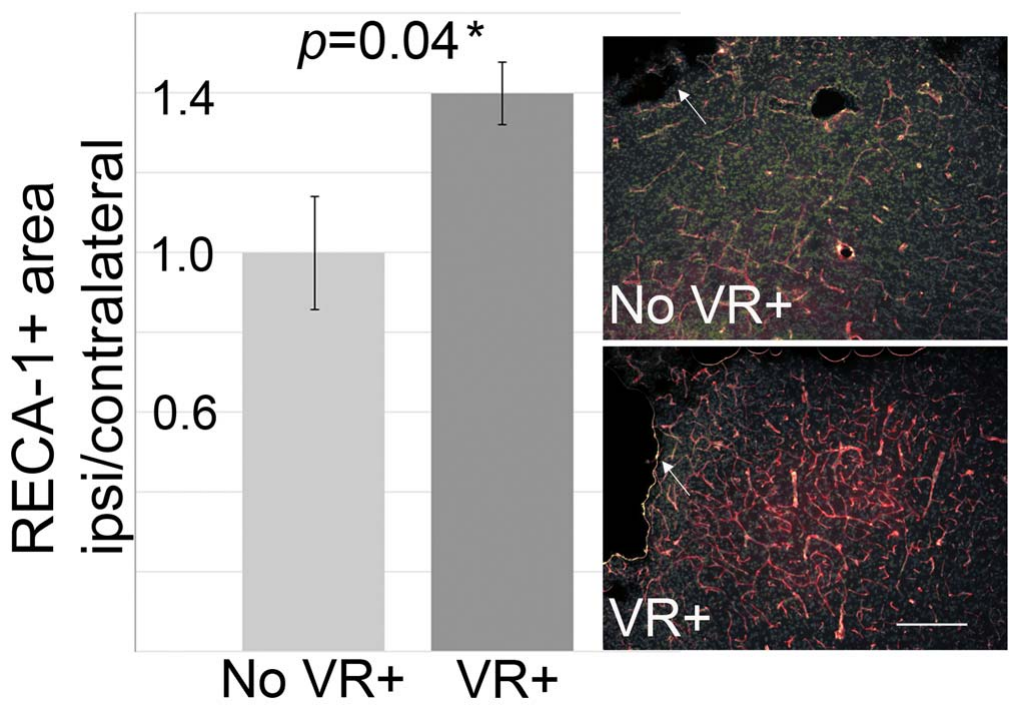
Increased density of blood vessels at perilesional regions with increased d14 vasoreactivity. RECA-1 immunofluorescence staining (red) with DAPI nuclear staining (green) compared in animals without (No VR+) and with (VR+) increased vasoreactivity on d14 (n = 4 per group). Corresponding subcortical regions close to the infarct on d14 showed a higher density of RECA-1 signal. One example per group is shown on the right. White arrows point to the lesion. White line indicates 100 µm.

To test if increased vasoreactivity was correlated to vascular anatomy in the border zone of infarction on d14, we analyzed the expression of the endothelial marker RECA. Subcortical regions at the borderzone of infarction showed a higher density of RECA - positive staining compared to the contralateral side in animals with increased vasoreactivity (Figure 6). Absolute area of staining (in mm^2^) was similar in both groups on the contralateral side (3.6+/−0.8 mm^2^ vs. 3.5+/−0.7 mm^2^).

## Discussion

Recanalization of the occluded vessel is highly correlated with a better functional outcome in patients affected by stroke [22], [23]. However, recanalization bears the risk of “reperfusion injury”, a phenomenon known and feared after surgical revascularization procedures, such as carotid endarterectomy [24], [25]. In early reports of post-stroke hyperperfusion, this phenomenon was suspected to be part of reperfusion injury; and a deleterious effect on stroke recovery was assumed [26]. In their studies of transient ischemia in cats, Heiss et al. found that the longer the duration of ischemia (30–120 minutes), the more severe and longer the immediate post-ischemic hyperperfusion, and the worse the chance of survival [5]. However, while these changes in CBF take place within the first minutes to hours after ischemia, not much is known about the late hyperperfusion phase observed here, occurring after days to weeks.

In clinical studies, depending on the time of analysis after stroke and the method used, between 10–50% of patients have areas of post-ischemic hyperperfusion [6], [7], [8], [10]. The fact that post-ischemic hyperperfusion could only be detected in some, but not all stroke patients, can be explained by recanalization that is partial or absent in some patients, as well as the transient nature of hyperperfusion which can be missed with a single examination. Kidwell et al. 2001 did not find a difference in clinical improvement in patients displaying hyperperfusion after stroke, but showed that tissue with post-ischemic hyperperfusion was very likely to become part of the final infarct.

In our study, late post-ischemic hyperperfusion between d1 and 14 was found in all rats, regardless of final lesion size. Similar to the findings of Kidwell et al., the area of hyperperfusion overlapped with the initial perfusion restriction, and even more, with final infarct volume.

The number of voxels with hyperperfusion was an indicator of infarct size. Small subcortical infarcts were characterized by an earlier onset of hyperperfusion (d1). In intermediate size cortico-subcortical infarcts, hyperperfusion was maximal on d4, but remittent on d14. Hyperperfusion was sustained and maximal on d14 in large hemispheric lesions. From such non-uniform temporal dynamics of blood flow adaptations it is difficult to extract a prognostic parameter at a single observation time point. However, an earlier occurrence and fewer affected voxels indicate a smaller final lesion. Although the number of hyperperfused voxels on d4 correlated to final infarct size, the correlation to functional test score on d14 was rather weak, which was most likely explained by our small sample size.

Based on our observations, we postulate that late post-ischemic hyperperfusion is a common part of the reperfusion cascade, and secondary to the initial ischemic impact.

Mechanistically, post-ischemic hyperperfusion may reflect a state of “vasoparalysis”, where endothelial cells within the affected region lose their autoregulatory capacity and remain in (sub)-maximal dilation. We tested this by analyzing the response to the vasodilatory stimulus CO_2_. We found that vasoreactivity within the ischemic area was depressed in most animals shortly after stroke, when hyperperfusion was maximal. Interestingly, we observed animals with an overshooting vasoreactivity on d14 after MCAO. These animals either had subcortical strokes with a rather low and constant number of hyperperfused voxels from d0 on, or intermediate cortico-subcortical lesions with a clear reduction of the hyperperfused area from d4 to d14. Such locally increased vasodilatory response to CO_2_ after ischemia-reperfusion has not been described before. In the series of events leading to CBF regulation after reperfusion, this probably reflects recovery of the vasculature from the vasoparalytic state. However, a limitation of our study is that arterial blood gases before and during the CO_2_ – challenge were not measured in the same set of animals subjected to longitudinal MRI, but in a separate group with identical settings used for MRI and anesthesia [17]. Our histological data, indicating a higher density of blood vessels in the vicinity of the infarct border in these animals, suggest that the high vasodilatory capacity might be due to young, still immature, blood vessels, which were formed in a compensatory reaction to facilitate supply to the peri-lesional tissue. Peri-lesional angiogenesis starts around 3 days post stroke; and blood vessel density has been shown to increase markedly after 7–15 days [27]. This has been associated with a higher peri-lesional CBF values [28].

Late post-ischemic hyperperfusion has been investigated after 30–90 minutes of MCAO in rats [29]. These authors also found a congruency between voxels with hyperperfusion and voxels that went on to be infarcted. The peak of hyperperfusion in their study was at 48 hours. Interestingly, they observed hyperperfusion in all animals after 30 minutes MCAO, but only in half of the 60 minute - and none of the 90 minute MCAO group. This is most likely due to the shorter observation span (only 24 hours in the 60 and 90 minute occlusion groups) in their study. As we show in our sample, larger infarcts have a later occurrence of hyperperfusion, so it was probably missed at 24 hours.

## Summary

In conclusion, late post-ischemic hyperperfusion was detected between d1 and 14 after transient ischemia in our rat model. It is likely that hyperperfusion observed in stroke patients is similar to this late post-ischemic hyperperfusion (in contrast to immediate or early post-ischemic hyperperfusion). Although voxels included in late post-ischemic hyperperfusion areas are likely to undergo infarction, the occurrence of hyperperfusion *per se* is not related to an unfavorable functional outcome. An early occurrence and smaller area of maximal hyperperfusion after stroke are associated with smaller lesions, while a late occurrence and larger area of hyperperfusion indicate a large infarct. Vasodilatory capacity can recover in former areas of hyperperfusion and even be above baseline at later time points, indicating recovery of vascular function or integration of newly formed blood vessels into the infarct border zone.
